# Large testicular adrenal rest tumours in a patient with congenital adrenal hyperplasia

**DOI:** 10.1530/EDM-14-0080

**Published:** 2015-02-01

**Authors:** J Rajkanna, S O Oyibo

**Affiliations:** Department of Endocrinology, Peterborough City Hospital Bretton Gate, Peterborough PE3 9GZ, UK

## Abstract

Testicular adrenal rest tumours (TARTs) are benign ACTH-dependent tumours that occur in males with congenital adrenal hyperplasia (CAH) and if left untreated can destroy testicular tissue. Corticosteroid suppressive treatment could result in the regression of these testicular tumours. We present a patient with bilateral large TARTs as a consequence of poor compliance to treatment and follow-up for his CAH, who consequently had to have bilateral orchidectomies and prosthesis replacement.

**Learning points:**

- TARTs are frequently seen in males with CAH, and can be misdiagnosed as primary testicular cancer.
- Patient compliance to treatment and follow-up are necessary to reduce the risk of testicular damage as a result of TARTs in patients with CAH.
- Boys with CAH should have periodic ultrasonographic screening from before adolescent age for early detection of TARTs.
- Regular monitoring of renin, 17-hydroxyprogesterone and androgens levels is required to assess corticosteroid suppressive treatment.
- Patients with CAH should be offered psychological support and information concerning CAH support groups.

## Background

Congenital adrenal hyperplasia (CAH) is an inherited disorder resulting from the deficiency of enzymes required for synthesis of cortisol in the adrenal gland. The commonest enzyme defect is 21-hydroxylase deficiency and the resultant cortisol deficiency leads to excessive corticotrophin (ACTH) production, which due to bypass effect results in excessive androgen production (1).

Adrenal rest cells first described in 1940 are a group of cells trapped within the developing gonad during foetal development (2). Usually <5 mm in size, they are found in the testis and surrounding tissue in 7.5–15% of new born and in about 1.5% of adults (3). Testicular adrenal rest tumours (TARTs) are benign ACTH-dependent tumours that have a reported prevalence of up to 94% in males with CAH (4). We present a patient with bilateral large TARTs as a consequence of poor compliance to treatment and follow-up.

## Case presentation

A 25-year-old male presented to the endocrine clinic in November 2011 with a history of tiredness, reduced libido and bilateral large testicles, which he wanted surgically removed (5).

He had been diagnosed with salt-losing CAH during his antenatal period and commenced on steroid replacement therapy soon after birth. However, he stopped taking all medications in 2000 because of family issues and although his testicles were felt to be lumpy in 2002, further investigation was hampered by poor compliance to treatment and follow-up. He did see the urologist in 2007 but failed to attend follow-up appointments and scans thereafter. He reappeared in the urology clinic in 2010 and again in 2011, after which he was re-referred to the endocrinology department by the urologist in readiness for testosterone replacement therapy after the proposed orchidectomy.

On examination he was well with normal secondary sexual features, but his testicles felt hard and three times the normal size. His serum testosterone level was 50.4 nmol/l. After the baseline investigations (see Table 1) and multiple non-attendances, he finally had bilateral orchidectomy and prosthesis replacement in October 2012. Further follow-up was difficult, and compliance with steroid replacement was again very poor.

**Table 1:** Results of blood tests pre- and post-surgery with laboratory reference values

**Biochemical test**	**Normal reference range**	**Pre-surgery December 2011**	**Post-surgery May 2013**	**August 2014**
17-OH-progesterone	<13 nmol/l	>152	>152	124
Testosterone	10–38 nmol/l	50.4	10.8	3.9
Corticotrophin (ACTH)	0–50 ng/l	139	–	–
Dehydroepiandrosterone	1.6–11 μmol/l	12	–	3.6
Androstenedione	1.4–9.1 nmol/l	–	–	21.0
Follicle-stimulating hormone	1–14 U/l	<1	–	3
Luteinizing hormone	1–9 U/l	<1	–	2
Prostate-specific antigen	<2.5 μg/l	0.27	–	0.76
0-min cortisol (pre-Synacthen)	–	327	–	–
30-min cortisol (post-Synacthen)	>550 nmol/l	354	–	–
Cortisol 2-h after morning hydrocortisone (10 mg)	–	–	–	734
Sodium	133–146 mmol/l	144	140	141
Potassium	3.5–5.3 mmol/l	4.2	4.3	4.5

–, Indicates test was not done.

## Investigation

Results of baseline and follow-up biochemical investigations are given in Table 1. The short Synacthen test demonstrated impaired adrenal gland function. The raised serum 17-hydroxyprogesterone, testosterone and dehydroepiandosterone levels were in line with the unsuppressed raised serum ACTH levels. Previous serum tumour markers (alphafetoprotein, lactate dehydrogenase and beta human chorionic gonadotrophin) were normal in 2010.

An ultrasound scan performed initially in 2010 confirmed large testicles (the right testis measured 6.2×4.7×2.6 cm, the left testis measured 6.6×3.9×3.6 cm and epididymis could not be visualised).

Pathological examination after orchidectomy demonstrated large testicles measuring 8.5×5×4 cm in size with no recognisable testicular parenchyma (stage 5) (3). The histology comprised sheets of cells separated by delicate fibrous septa with polygonal tumour cells with abundant oeosinophilic cytoplasm, round, variably sized nuclei and prominent central nucleoli. This histology report is the characteristic of TARTs. The report also mentioned the possible presence of some Reinke crystals.

## Treatment

His initial treatment (restarted in 2011) was hydrocortisone 5 mg twice a day for steroid replacement therapy with the addition of dexamethasone 0.25 mg at bedtime for ACTH suppression.

## Outcome and follow-up

He failed to attend follow-up appointments or do any blood tests after his operation, despite multiple attempts to contact him. However he was discovered again in June 2014 when he was admitted to the Emergency Department with a history of vomiting, dehydration and adrenal insufficiency. He had not been taken dexamethasone, but assured us that he had been taking hydrocortisone tablets. He was discharged on hydrocortisone 10 mg twice a day. We were finally able to obtain some more blood samples (see Table 1) and we await further discussion concerning ACTH suppression and testosterone replacement.

## Discussion

Although TARTs are usually benign, they obstruct the seminiferous tubule, leading to testicular destruction and failure if untreated. There are five stages (stage 1: adrenal rest cells are present within the rete testis; stage 2: hyperplasia and hypertrophy of the rest cells; stage 3: the rest cells compress rete testis; stage 4: induced fibrosis and lymphatic infiltration of testicular perenchyma; stage 5: irreversible damage of testicular perenchyma). They are usually bilateral, with a reported prevalence of up to 94% in males with CAH and are ACTH dependant. As a result of 21-hydroxylase deficiency these patients have high serum ACTH, 17-hydroxyprogesterone and androgens. Intensive corticosteroid therapy, which suppresses ACTH secretion, often leads to a reduction in size of these tumours (4) (6). If glucocorticoid treatment is ineffective, testis-sparing orchidectomy is recommended to save unaffected testicular parenchyma (7).

It can be difficult to distinguish between TARTs and Leydig cell tumours of the testis and there have been reports of the coexistence of both conditions in patients with CAH (8). However, Leydig cell tumours are rarely bilateral and are not corticosteroid-responsive like TARTs. In addition, the lack of malignant features on histology after many years of diagnosis makes Leydig cell tumour less likely. Our patient did have very high serum testosterone levels, which has been reported as more suggestive of the presence of a Leydig cell tumours (8). There was also a histological suspicion of the presence of some Reinke crystals, which are cytoplasmic rod-like crystalloids particles found in 30–46% of the Leydig cell tumours. The possible presence of a few of these crystals in our case could indicate the co-existence of benign Leydig cell tumours (9). In addition, the fall in serum testosterone back to the lower end of normal reference range post-surgery does potentiate the suspicion of the possible co-existence of functioning Leydig cells. Unfortunately, because of the poor follow-up the testosterone issue could not be further assessed. In addition, the steroid-responsiveness of TARTs could not be assessed in this patient with large destructive tumours.

For patients with stage 4 TARTs, where medical therapy (corticosteroid suppression) has failed to result in tumour regression, testis-sparing surgery is the treatment of choice to prevent progression to stage 5 when severe obstructive fibrosis of the testicular parenchyma ensues (3) (7). Testicular biopsy to assess the presence of healthy testicular parenchyma should precede surgery. Our patient had already reached stage 5 with very large testicular masses which were causing discomfort, both physically and psychologically. The patient opted for bilateral total orchidectomies and replacement prosthesis. Pathological examination revealed the absence of any testicular parenchyma.

There is bound to be psychosocial issues associated with the affectation of testicular anatomy and function in patients with CAH and TARTs. These can range from depression to impairment of sexual well-being (10). A population-based survey of patients with CAH in Norway demonstrated that they had significantly impaired subjective health status as measured by the SF-36 questionnaire, and a higher proportion of the patients than the general population receiving working disability benefit (11). These could be major contributory factors to this patient's poor compliance with treatment and follow-up during childhood and adolescence. The importance of early introduction of patients with CAH to a child psychologist and available CAH support groups cannot be overemphasised.

In conclusion, we have described a patient with CAH who developed large TARTs as a result of poor compliance to medical treatment and follow-up, and subsequently required bilateral orchidectomies and replacement prosthesis. We reiterate that adequate medical treatment, regular outpatient follow-up and psychosocial support are required by patients with CAH to prevent acute and chronic, physical and psychological complications.

## Patient consent

Written informed consent has been obtained from the patient for publication of the submitted article and any accompanying images. The signed copy of the consent form has been provided.

## Author contribution statement

The first author (Dr J Rajkanna) and the second author (Dr S O Oyibo) identified the patient, wrote up the case and presented the case at the Society for Endocrinology BES 2014 Meeting. Dr S O Oyibo is also the named physician of the patient.
